# The Impact of Dual-Wavefront Propagation of Electromagnetic Waves in Bio-Tissues on Imaging and In-Body Communications

**DOI:** 10.3390/bios15100667

**Published:** 2025-10-03

**Authors:** Lei Guo, Kamel Sultan, Fei Xue, Amin Abbosh

**Affiliations:** School of EECS, The University of Queensland, St Lucia, QLD 4072, Australia; l.guo3@uq.edu.au (L.G.); f.xue@uq.edu.au (F.X.); a.abbosh@uq.edu.au (A.A.)

**Keywords:** electromagnetic (EM) propagation, medical imaging, in-body communication, EM travel time, EM imaging

## Abstract

Understanding how electromagnetic (EM) waves travel through different tissues is important for EM medical imaging, sensing, and in-body communication. It is known that EM waves in lossy bio-tissues are nonuniform and do not strictly follow the least time or least loss paths. Instead, they exhibit two distinct wavefronts: the phase wavefront and the amplitude wavefront, which are generally oriented at different angles. The impact of that on imaging and in-body communications is investigated and validated through comprehensive analysis and full-wave EM simulations. Additionally, the impact of a matching medium, commonly used to reduce antenna–skin interface reflections in medical EM applications, on the direction of EM wavefronts, travel time, phase changes, and attenuation is analyzed and quantified. The results show that the Fermat principle of least travel time, often used to estimate EM wave travel time for localization in medical imaging and wireless endoscopy, is only accurate when the loss tangent or dissipation factor of both the matching medium and tissues is very low. Otherwise, the results will be inaccurate, and the dual wavefronts should be considered. The presented analysis and results provide guidance on EM wave travel time and the direction of phase and amplitude wavefronts. This information is valuable for developing reliable processing algorithms for sensing, imaging, and in-body communication.

## 1. Introduction

The rapid advancement in the design and development of medical devices that use electromagnetic (EM) waves has significantly heightened interest in understanding how these waves interact with biological tissues [1,2,3,4]. This understanding is vital for optimizing the performance and reliability of both diagnostic and therapeutic systems, including implantable devices, electromagnetic imaging frameworks, and in-body communication systems [5,6]. Accurate knowledge of EM wave propagation behavior within complex, heterogeneous, and inherently lossy biological tissues directly influences power transfer efficiency, the accuracy of medical imaging, and the fidelity of signal transmission for in-body communications.

In medical electromagnetic imaging, this understanding directly impacts the accuracy of reconstructed images [2,7,8,9], enabling better detection and localization of anomalies such as tumors, fatty liver disease, or ligament injuries [8,10,11,12,13,14]. Additionally, microwave algorithms used in medical imaging and diagnostics rely on accurate wave propagation velocity and path estimations for correct data interpretation and image reconstruction [15]. Furthermore, precise modeling of wave behavior can significantly reduce the number of training cases required for data-driven imaging algorithms, streamlining their implementation and reducing computational complexity.

Beyond imaging, modeling of EM wave propagation is essential for therapeutic applications such as microwave ablation and hyperthermia therapy. Understanding the EM wave path helps determine the required power levels and optimize the placement of sensors or antennas to achieve targeted tissue heating with minimal damage to surrounding tissues [16]. Similarly, for wireless power transfer and communication with implantable devices such as pacemakers, insulin pumps, or wireless capsule endoscopes, accurate EM propagation modeling ensures efficient and reliable energy and data transmission [17,18,19,20]. This is particularly critical for in-body environments where highly variable dielectric properties, tissue interfaces, and complex geometries influence wave behavior in non-intuitive ways [21].

Extensive research in localization and sensing has been performed to address these complexities [4,5,22,23,24,25,26,27,28]. The outcomes of that research offer promising solutions for applications like wireless capsule endoscopy, where conventional methods are impractical for the small bowel or continuous long-term monitoring. Techniques exploiting variations in signal attenuation, relative permittivity, and multipath effects are being explored to estimate the positions and trajectories of in-body devices. However, most existing localization algorithms still rely on simplified path loss and time-of-flight models, which inadequately capture the intricate propagation dynamics in heterogeneous, lossy biological environments.

Traditionally, EM wave paths within biological tissues have been estimated using Fermat’s principle of least time [29,30]. Snell’s law, derived from Ibn Sahl and Fermat’s principle of least time, dictates how EM waves bend when transitioning between different media [31,32]. Fermat’s principle indicates that EM waves take the path that minimizes travel time between two points. This path may not be the shortest geometric distance, but the least travel time path. This is true in lossless media [33,34]. However, biological tissues are inherently lossy. In such lossy media, the dielectric properties and propagation constant become complex, incorporating real and imaginary components [35]. When an electromagnetic wave enters a lossy medium, it exhibits two distinct wavefronts: the phase wavefront, representing surfaces of constant phase, and the amplitude wavefront, representing surfaces of constant amplitude. This characteristic, known as the dual-wavefront property, has long been recognized in electromagnetics as a feature of inhomogeneous (or nonuniform) plane waves. Therefore, Fermat’s principle is not complete to determine the wave propagation path in a lossy medium, e.g., the bio-tissues. The divergence between phase and attenuation angles in lossy media has long been recognized within the framework of inhomogeneous plane waves [36,37,38,39]. While these studies established the theoretical foundation, the implications of this dual-wavefront phenomenon arising from lossy tissues, on medical EM imaging, sensing, and in-body communications remain largely unexplored.

**Figure 1:**
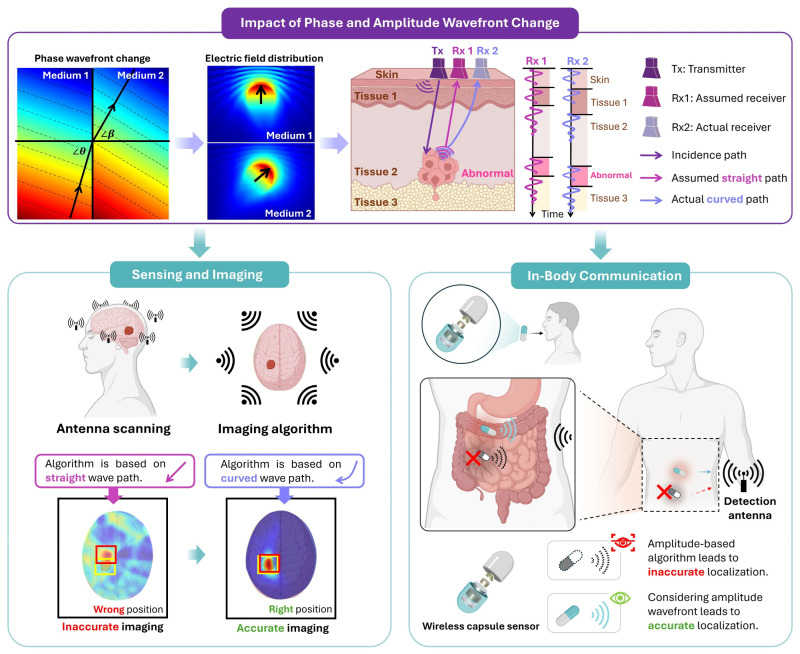
A conceptual diagram to demonstrate the impact of EM phase and amplitude wavefronts in lossy tissues on sensing, imaging and in-body communication.

To address this issue, theoretical analysis and comprehensive simulations are used to demonstrate that the wave propagation path derived from phase distribution, i.e., phase wavefront, is primarily defined by the dielectric constant of the media, while conductivity has a minor impact on it. On the other hand, the wave path based on amplitude distribution, i.e., the amplitude wavefront, varies significantly with changes in media conductivity. This distinction has critical implications for in-body communication and medical EM imaging. For example, inaccurate estimation of the signal travel time and loss may result in significantly distorted sensing and tracking results, as conceptualized in Figure 1. While localization algorithms using phase information to estimate wave travel time can provide robust tracking accuracy of, for example, wireless endoscopes, amplitude-based approaches may not adhere to Fermat’s principle. Consequently, the wave propagation path based on the amplitude wavefront should be derived, which is primarily determined by the conductivity of the lossy medium. The subsequent sections of this paper present the theoretical part, simulation results, and a quantitative assessment with a focus on medical electromagnetic imaging and in-body communications.

## 2. Dual-Wavefront Electromagnetic Propagation in Bio-Tissues

Assume a homogeneous, isotropic, time-invariant, linear, and source-free medium with a permittivity ε, conductivity σ, and permeability μ. The steady-state solution for the EM wave in such a medium can be calculated as [37,40]:

(1) $$E=E_{o}e^{-\gamma \cdot r},H=H_{o}e^{-\gamma \cdot r}$$

where $E_{o}$ and $H_{o}$ are constant complex vectors, r is a position vector, and γ=α+jβ is the complex propagation constant vector comprising the attenuation vector α and the phase vector β, while r is the position vector. To avoid having a trivial solution, the complex propagation constant vector should meet the following conditions [37]:

(2) $$\gamma \cdot \gamma =\gamma_{o}^{2}$$

where $\gamma_{o}$ is the intrinsic propagation constant of the medium expressed as

(3) $$\gamma_{o}=\alpha_{o}+j\beta_{o}$$

and $\alpha_{o}$ and $\beta_{o}$ are the intrinsic attenuation and phase constants of the medium [37]:

(4) $$\begin{array}{c} \alpha_{o}=\omega \sqrt{\frac{\mu \varepsilon }{2}\left[\sqrt{1+{\left(\frac{\sigma }{\omega \varepsilon }\right)}^{2}}-1\right]}\\ \beta_{o}=\omega \sqrt{\frac{\mu \varepsilon }{2}\left[\sqrt{1+{\left(\frac{\sigma }{\omega \varepsilon }\right)}^{2}}+1\right]} \end{array}$$

**Figure 2:**
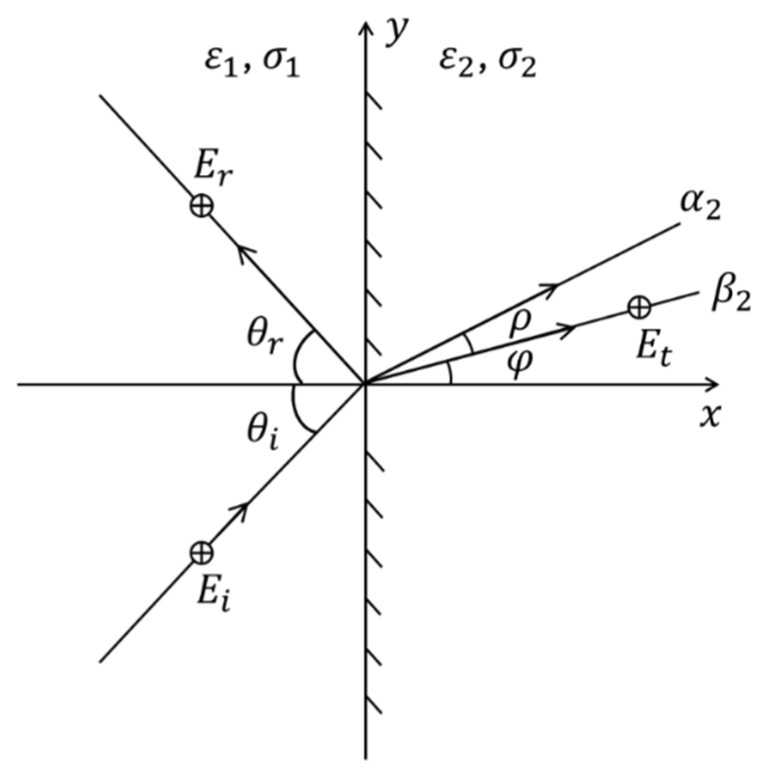
A uniform ${TM}_{z}$ plane wave incident on an interface between two lossy media.

Without losing the generality of the analysis, consider a ${TM}_{z}$ polarized wave (commonly used in microwave medical imaging and sensing applications [8,11,12]), at the incident plane of an interface between two lossy media (media 1 and 2) as shown in Figure 2. The incident field $E_{i}$ and refracted field $E_{t}$ can be expressed as [33]:

(5) $$E_{i}=\hat{z}E_{i,z}e^{\left[-\alpha_{o1}\left(x\cos\theta_{i}+y\sin\theta_{i}\right)-j\beta_{o1}\left(x\cos\theta_{i}+y\sin\theta_{i}\right)\right]}$$

(6) $$E_{t}=\hat{z}E_{t,z}e^{\left[-\alpha_{2}\left(x\cos\left(\varphi +\rho \right)+y\sin\left(\varphi +\rho \right)\right)-j\beta_{2}\left(x\cos\varphi +y\sin\varphi \right)\right]}$$

According to (2), (3) and (6), it is possible to derive the following [39]:

(7) $$\alpha_{2}^{2}-\beta_{2}^{2}=\alpha_{o2}^{2}-\beta_{o2}^{2};\alpha_{2}\beta_{2}\cos\rho =\alpha_{o2}\beta_{o2}$$

In addition, by applying Snell’s law [38] for both the attenuation and phase constant angles at the interface of the two media, it is possible to show that

(8) $$\beta_{o1}\sin\theta_{i}=\beta_{2}\sin\varphi ;\;\alpha_{o1}\sin\theta_{i}=\alpha_{2}\sin\left(\varphi +\rho \right)$$

Combining (7) and (8), the attenuation and phase vectors of medium 2 can be calculated as

(9a) $$\alpha_{2}=\sqrt{\frac{{\left|\gamma_{o1}\right|}^{2}\sin^{2}\theta_{i}+Re\left(\gamma_{o2}^{2}\right)+\left|\gamma_{o1}^{2}\sin^{2}\theta_{i}-\gamma_{o2}^{2}\right|}{2}}$$

(9b) $$\beta_{2}=\sqrt{\frac{{\left|\gamma_{o1}\right|}^{2}\sin^{2}\theta_{i}-Re\left(\gamma_{o2}^{2}\right)+\left|\gamma_{o1}^{2}\sin^{2}\theta_{i}-\gamma_{o2}^{2}\right|}{2}}$$

where $\gamma_{o1}=\alpha_{o1}+j\beta_{o1}$ and $\gamma_{o2}=\alpha_{o2}+j\beta_{o2}$. If we assume the EM field in medium 1 to be uniform, which is the case in medical EM sensing, imaging, and in-body communication, where the used antennas generate a uniform field, then $\alpha_{o1}=\alpha_{o}$ and $\beta_{o1}=\beta_{o}$.

The refracted wave exhibits two distinct wavefronts: an amplitude wavefront, corresponding to surfaces of constant amplitude, and a phase wavefront, corresponding to surfaces of constant phase. These wavefronts do not represent two separate waves but rather two geometric characteristics of a single inhomogeneous plane wave in a lossy medium. Accounting for these two directions is essential for accurate medical EM imaging and in-body communications. To illustrate this, two media are modeled in COMSOL Multiphysics 5.3a with an incident angle of 45° at 1 GHz, a frequency commonly used in biomedical microwave imaging due to its balance between tissue penetration and spatial resolution [41,42,43,44,45,46].

**Figure 3:**
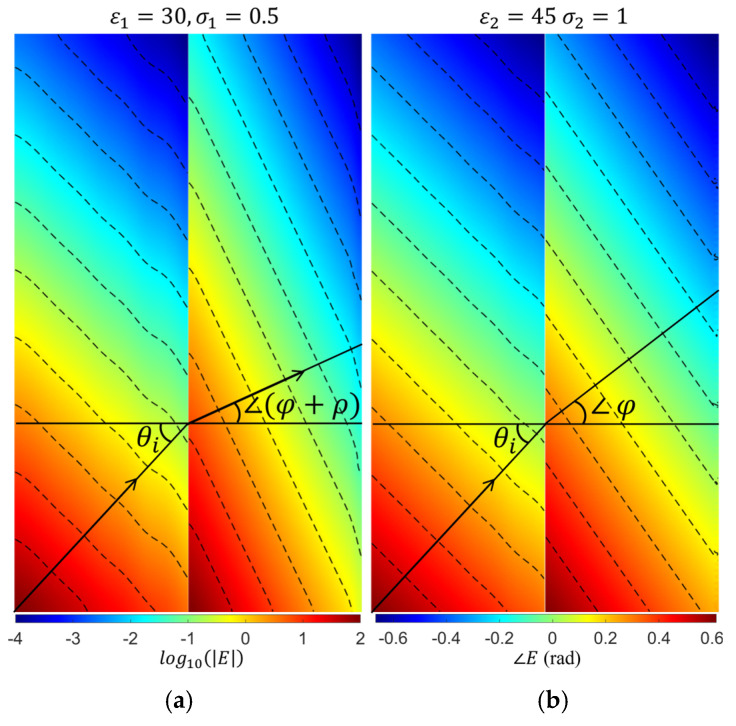
(**a**) The amplitude and (**b**) phase distributions, along with the contour lines that were used to extract the refractive attenuation angle (φ+ρ) and phase angle (φ). The amplitude distribution is shown in a logarithmic scale, and the phase distribution is shown in radians.

In the ComSoL simulations, the directions of the amplitude and phase wavefronts in both media are extracted from the contour of the electric field amplitude and phase distributions. Figure 3 shows an example of those distributions with their contours, where the relative permittivity and conductivity are (30, 0.5 S/m) and (45, 1 S/m) for media 1 and 2, respectively.

**Figure 4:**
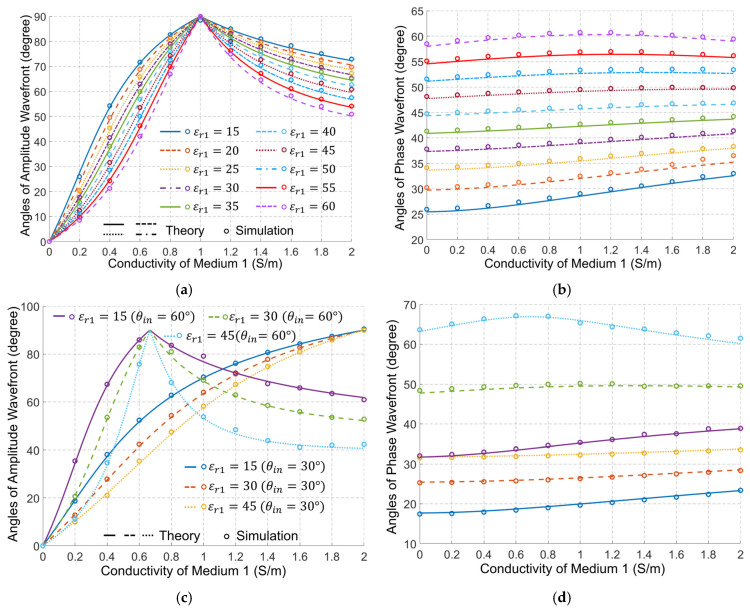
Angles of the amplitude wavefront (the left column) and phase wavefront (the right column), as a function of medium#1 properties ($\varepsilon_{r1}$, $\sigma_{1}$) at 1 GHz when $\theta_{i}=45{}^{\circ}$ (**a**,**b**), and $\theta_{i}=30{}^{\circ}$ and 60° (**c**,**d**). The properties of medium#2 are fixed at the average properties of the human body, $\varepsilon_{r2}=40$, $\sigma_{2}=0.5$ S/m. Note: The legend of (**b**) and (**d**) is the same as in (**a**) and (**c**), respectively.

Figure 4 shows the theoretically calculated angles of the amplitude and phase wavefronts for a wide range of cases. In those calculations, we fixed the dielectric properties of medium#2 to be the average properties of the human body, whereas the properties of medium#1 are changed to emulate using different types of matching medium between the external antennas and bio tissues. The results show a perfect match between the theory and simulations. As an initial verification of the results in Figure 4, it is clear that when the properties of medium#1 are the same as the properties of medium#2, $\varepsilon_{r1}=\varepsilon_{r2}=40$, ${\sigma_{1}=\sigma \;}_{2}=0.5$ S/m, the two media will be effectively one, and thus the transmitted wave in medium#2 has the same wavefront direction as the incident wave.

Figure 4 indicates that the dielectric properties of medium#1 have a significant effect on the direction of the amplitude wavefront in medium#2. Increasing the conductivity in medium#1 increases the angle of the amplitude wavefront till it becomes 90°, which indicates an amplitude variation along the interface between the two media, and thus a strong surface wave. After that, increasing the conductivity of medium#1 gradually reduces the angle of the amplitude wavefront. On the other hand, increasing the permittivity of medium#1 decreases the angle of the amplitude wavefront for the waves in medium#2.

Figure 4a shows two distinct points: When the conductivity of medium# 1 equals zero, i.e., a lossless matching medium, and when the conductivity of medium#1 is 1 S/m. When medium#1 is lossless, we can verify from (4) that the attenuation constant for medium#1 is zero, and thus we can conclude from (8) that the only solution for the attenuation constant in medium#2 is φ+ρ =0° indicating an amplitude wavefront in medium#2 that has an angle of zero. Thus, its direction is normal to the interface between the matching medium and the human body, indicating an amplitude variation of the transmitted wave in medium#2 at the normal direction to the interface. For the other distinct point, it is possible to verify that when the conductivity of medium#1 is 1 S/m, the solution of (9a) gives an imaginary value for the attenuation constant, indicating an amplitude wavefront of angle 90°, i.e., along the interface between the matching medium and the human body. This is a mathematical property of the expression that corresponds physically to the condition where the tangential phase constant in medium#1 equals the intrinsic wavenumber of medium#2. Figure 4b shows that changes in the conductivity of medium# 1 have a small but important effect on the direction of the phase wavefront in medium 2. Increasing the conductivity of medium#1 increases the angle of the phase wavefront to a specific limit. After that, increasing the conductivity reduces that angle. On the other hand, increasing the permittivity of medium#1 increases the angle of the phase wavefront in medium#2. It is obvious, though, that the permittivity has a much larger effect on the angle of the phase wavefront. Figure 4c,d show that the phenomenon illustrated above is not affected by a change of the incident wave angle, except for a shift of the value of the conductivity of medium#1 at which the amplitude wavefront vector becomes along the interface between two media. A decrease in the incident angle leads to an increase in that value.

Since medium#1 in medical EM sensing, imaging, and in-body communications is a matching medium placed between the external antennas and the human body, the dielectric properties of this medium significantly impact the performance of these systems. Traditionally, the design and selection of the matching medium have been based on the value of the reflection coefficient of the external antennas [47,48]. However, this factor alone is not sufficient. Figure 4 shows that changing the properties of the matching medium changes the direction of EM wave propagation within the human body. Since all sensing, imaging, and in-body communications target a specific region inside the human body, the direction of the EM wavefronts must be a crucial consideration in the design of the matching medium and related systems.

## 3. Results and Discussions

This section presents two representative biomedical applications to demonstrate the practical implications of the dual-wavefront behavior of EM waves in lossy biological media. The first application focuses on microwave medical imaging, where the direction of the amplitude wavefront critically influences signal detection and image reconstruction. The second application examines microwave in-body communication, particularly the use of signal phase and amplitude information for accurate localization in wireless capsule endoscopy. For both cases, full-wave simulation results are provided to validate the theoretical predictions and quantify the impact of conductivity and permittivity variations on wavefront behavior, travel time, and signal distribution. These findings highlight the importance of considering both phase and amplitude directions in the design and analysis of EM-based biomedical systems. The dielectric properties used in this work were selected to reflect the range of different values of biological tissues within the frequency range of 0.5–4 GHz, which is the most widely used frequency range for electromagnetic biomedical applications, as summarized in Table 1. The simulation geometries were chosen to reflect realistic biomedical scenarios. For instance, the target radius is comparable to the size of tumors, cysts, strokes, or vascular structures of clinical interest in microwave imaging. Likewise, the coupling region thickness, organ dimensions (e.g., head diameter ~15–20 cm, knee joint cross section ~12 cm, and torso diameter ~20–40 cm), and receiver spacing are consistent with those used in experimental microwave imaging systems [8,12]. These considerations ensure that the theoretical and numerical results presented here are directly relevant to biomedical applications.

### 3.1. EM Imaging

**Figure 5:**
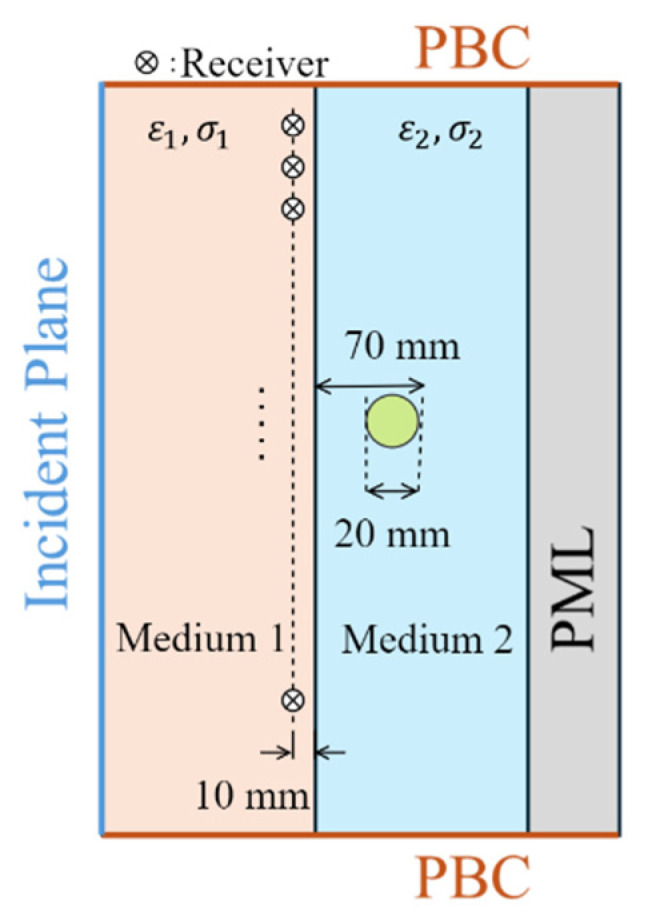
Simulated scenario that approximates EM biomedical sensing and imaging. PML: perfect matched layer. PBC: periodic boundary condition.

Medical EM imaging is used to detect and locate abnormal tissues within the human body using antennas placed outside the body, usually in contact with the skin via a matching or coupling medium. Figure 5 illustrates a basic example of EM sensing. In this scenario, two different media are considered: the matching medium (medium#1) and the biological tissues (medium#2). The matching medium is used to couple the imaging antennas with the tissues, improving the penetration of EM waves into the tissues. This medium helps to enhance the accuracy and effectiveness of detecting abnormal tissues. To emulate an abnormal tissue, such as a tumor, a circular target, which has a radius of 10 mm, with its center located 50 mm from the interface between the two media, is embedded in medium#2. The target has a relative permittivity of 70 and a conductivity of 2.5 S/m. The relative permittivity of medium#1 ($\varepsilon_{1}$) and medium 2 ($\varepsilon_{2}$) are assumed to be 30 and 45, respectively. The conductivity of medium#2 ($\sigma_{2}$) is 1 S/m, while the conductivity of medium#1 ($\sigma_{1}$) varies from 0 to 0.9 S/m. A ${TM}_{z}$− and a TE− polarized plane wave incident from the left side of medium 1 with an incident angle of 60°. The receivers are positioned along a line in medium#1, located 10 mm from the interface between the two media. The electric field distribution of the target response $E_{s}$ (scattered field) in both media#1 and 2 is defined as

(10) $$E_{s}=E_{ref}-E_{target}$$

where $E_{ref}$ is the electric field distribution when the target is absent in medium#2, and $E_{target}$ is the electric field distribution when the target is present in medium#2.

The derived theory implies that variations in $\sigma_{1}$ and $\sigma_{2}$ significantly affect the angle of $\alpha_{2}$, which represents the direction of the amplitude wavefront in medium#2. As shown in Figure 4a, the amplitude wavefront direction varies with changes in $\sigma_{1}$. This inference is particularly important in medical EM imaging, where a matching medium is usually used as a background medium, i.e., medium#1. Therefore, changes in the conductivity of that medium eventually result in variations in the amplitude distribution of the received signals by an antenna array external to the body.

**Figure 6:**
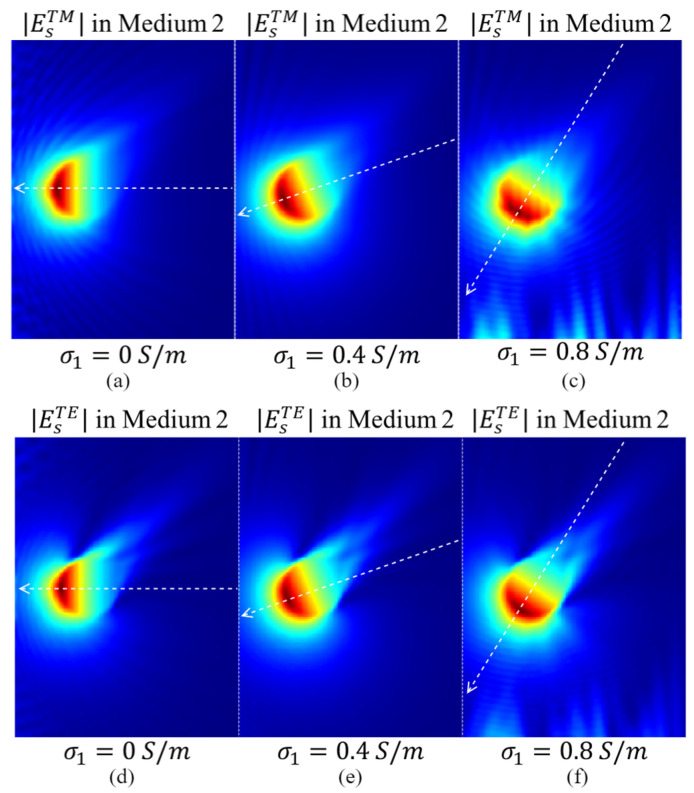
The amplitude distributions of $E_{s}^{TM}\;$ in medium#2 for different conductivities of medium#1: (**a**) 0 S/m, (**b**) 0.4 S/m, and (**c**) 0.8 S/m. (**d**–**f**) show the amplitude distributions of $E_{s}^{TE}\;$ in medium#2 for different conductivities of medium#1 (0 S/m, 0.4 S/m, and 0.8 S/m, respectively).

The simulated amplitude distributions of $E_{s}$ under the *TM* ($E_{s}^{TM}$) and *TE* ($E_{s}^{TE}$) polarizations using the configuration shown in Figure 5 with different values of $\sigma_{1}$ are presented in Figure 6. The amplitude wavefront directions of $E_{s}^{TM}$ and $E_{s}^{TE}$ vary significantly with changes in $\sigma_{1}$. When medium#1 is lossless, the amplitude wavefronts of $E_{s}^{TM}$ and $E_{s}^{TE}$ in medium#2 are normal to the interface between the two media. Consequently, the receiver closest to the target (the receiver in the middle of Figure 6) detects the signal with the highest intensity. As $\sigma_{1}$ increases, $E_{s}^{TM}$ and $E_{s}^{TE}$ amplitude wavefronts shift toward the side of medium#2, altering the distribution of the received signals. Consequently, the receiver with the highest signal intensity shifts from the middle position to those closer to the bottom of medium#2.

**Figure 7:**
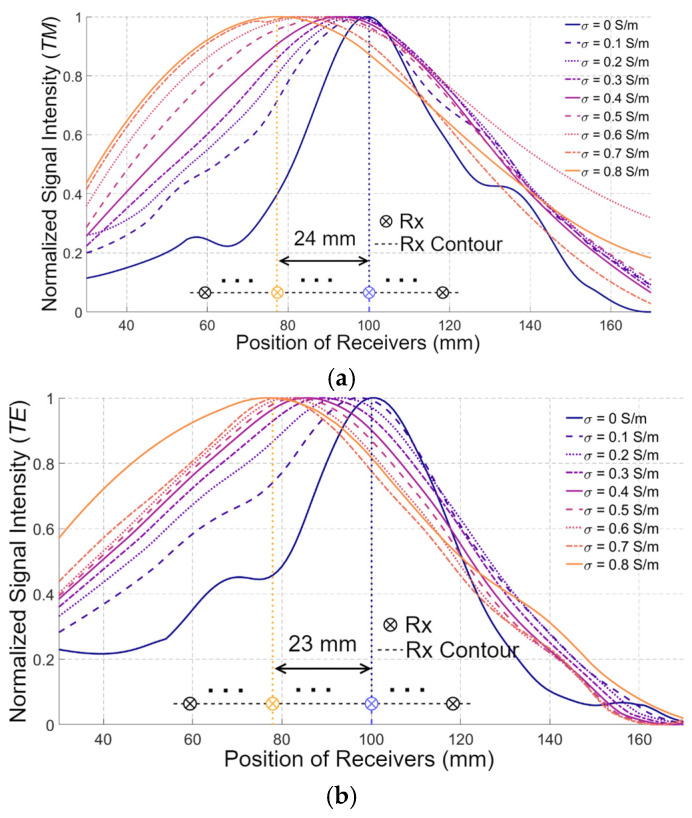
The amplitudes of $E_{s}^{TM}$ (**a**) and $E_{s}^{TE}$ (**b**) collected at the receiver (Rx) positions, when $\sigma_{1}$ varies from 0 S/m to 0.9 S/m.

Figure 7 presents the amplitudes of $E_{s}^{TM}$ and $E_{s}^{TE}$ collected at the receivers with the positions shown in Figure 5 for different values of $\sigma_{1}$. When medium#1 is lossless, the receiver near the middle of the measurement contour, i.e., the one closest to the target, exhibits the highest signal intensity for both $E_{s}^{TM}$ and $E_{s}^{TE}$ polarizations, as expected. However, as $\sigma_{1}$ increases, the receiver with the highest signal intensity becomes closer to the lower side of media #1 and 2.

This phenomenon is presented in both $E_{s}^{TM}$ and $E_{s}^{TE}$ polarizations. For example, when $\sigma_{1}$ increases to 0.9 S/m, the receiver with the maximum signal intensity shifts to the one located 24 mm and 23 mm away from the middle receiver in *TM* and *TE* polarizations, respectively. Assuming the distance between the adjacent receivers is 5 mm and denoting the receiver near the bottom of the medium as #1, the presence of the maximum signal intensity shifts from the 20th receiver to the 15th receiver. Those results confirm the wrong conventional assumption of a fixed wavefront for the EM waves when changing the properties of the matching medium.

These results are particularly important for EM imaging algorithms that rely on data-driven approaches. For example, a pure data-driven algorithm trained on data collected from scenarios with a lossless matching medium may identify a pattern where the target is located near the receiver with the highest signal intensity. Consequently, the trained model may generate incorrect sensing or imaging results when applied to data collected from scenarios with a lossy matching medium, where the receiver with the highest signal intensity is located farther from the target. The results also demonstrate the importance of developing physics-guided data-driven algorithms for EM sensing and imaging, which use the physical rules, such as the one presented in this work, to guide the training process.

### 3.2. In-Body Communication

In-body communication is another important application significantly affected by EM wave propagation in a lossy medium, e.g., bio-tissues. The in-body communication system aims to track the position of the in-body transmitting antenna using signals collected by receiving antennas outside the body. Two frequency bands are commonly used in in-body communication based on the Medical Implant Communication Service: around 400 MHz and 2.3 GHz [52]. While the low-frequency band provides desirable transmission efficiency, the higher-frequency band enables more accurate localization and reduced antenna size. One challenge of using the high frequency (2.3 GHz) is the significantly increased reflection at the interface between the in- and out-body regions, caused by the impedance mismatch between them. A matching medium is usually used between the external antenna and the body to alleviate the reflection. In the following investigations, the relative permittivity of the matching medium located in the outer-body region ($\varepsilon_{1}$) ranges from 15 to 60, while the conductivity ($\sigma_{1}$) varies from 0 S/m to 2 S/m, corresponding to the values used in matching media for medical EM imaging [53].

**Figure 8:**
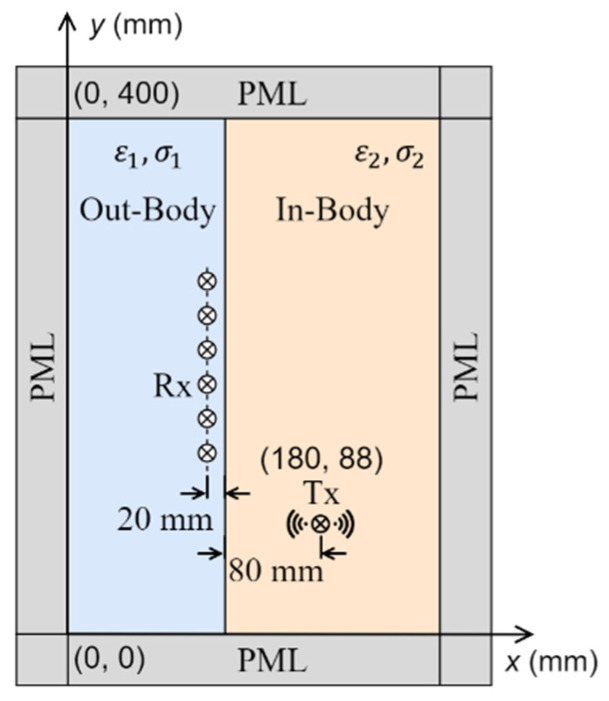
Simulated scenario of in-body communication, where a transmitting antenna is placed inside the body and an array of receiving antennas is placed outside the body.

A simplified scenario of in-body communication is shown in Figure 8. The simulation domain includes two regions, the in-body and the out-of-body regions. A transmitting antenna, emulating a capsule used for wireless endoscopy, is placed inside the body region, 80 mm from the skin, and 10 receiving antennas are evenly distributed in the out-of-body region, 20 mm from the interface. Without losing the generality of the conclusions, the in-body region is assumed to have a relative permittivity ($\varepsilon_{2}$) of 40 and a conductivity ($\sigma_{2}$) of 0.5 S/m. Two main approaches are widely used to track the position of the transmitting antenna: one of them is based on signal amplitudes, while the other uses the phase of the received signals [5], or signal travel time, which has been conventionally estimated using Fermat’s principle.

**Figure 9:**
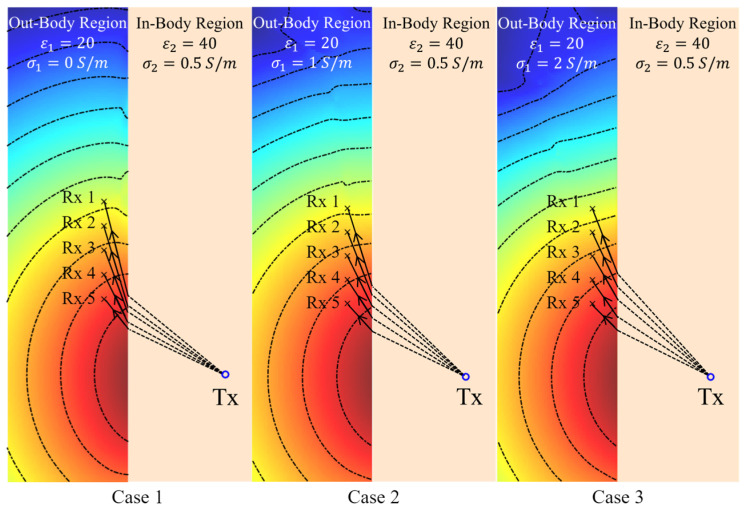
Theoretical phase wavefront calculated using the phase distribution of the electric field at 2.3 GHz in medium#1 (matching medium) when the conductivity ($\sigma_{1}$) of that medium varies from 0 S/m to 1 S/m.

**Figure 10:**
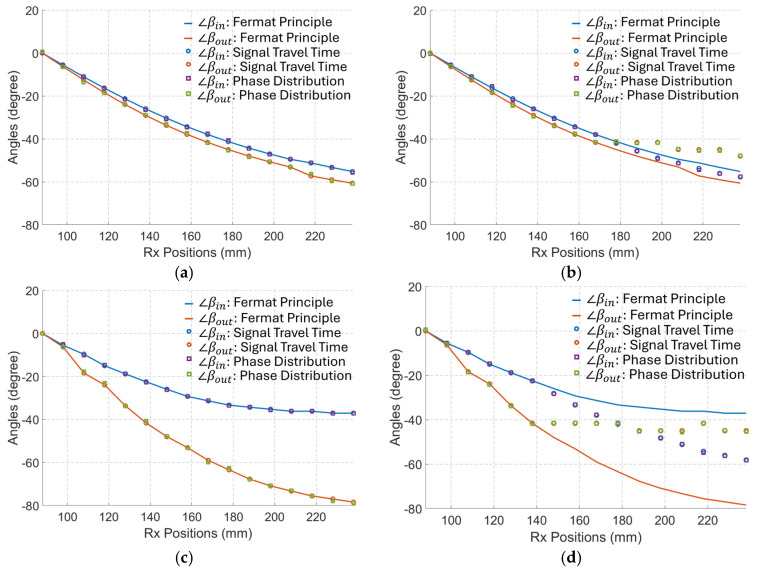
The calculated incident angles ($∠\beta_{in}$) in the in-body region and the refractive angles (∠$\beta_{out}$) in the out-body region for each of the Rx based on the Fermat Principle, the signal time delay, and the phase distribution. (**a**) When $\varepsilon_{1\;}=\;35,\;\;\sigma_{1}\;=\;0$ S/m. (**b**) When $\varepsilon_{1}\;=\;35,\;\;\sigma_{1\;}=\;2$ S/m. (**c**) When $\varepsilon_{1\;}=\;15,\;{\;\sigma }_{1}\;=\;0$ S/m. (**d**) When $\varepsilon_{1}\;=\;15,\;\;\sigma_{1}\;=\;2$ S/m.

Based on the previously presented results in Figure 4, variations in $\sigma_{1}$ have a small influence on the angles of the phase wavefront in the in-body region when $\varepsilon_{1}$ is large, i.e., a small dissipation or loss factor, defined as $\left(\sigma /\omega \varepsilon \right)$. In agreement with that conclusion, Figure 9 (Cases 1 and 2) shows that $\sigma_{1}$ has a minor impact on the phase wavefront direction at 2.3 GHz when the dissipation factor is very small. However, when the conductivity is large, or more importantly, when the dissipation factor is relatively large, the phase wavefront changes significantly with the dielectric properties as shown in Figure 9 (Case 3). Consequently, the phase wavefront determined by the Fermat principle, COMSOL simulations (the least travel time), and the phase wavefront direction should remain reasonably close to each other with changes in $\sigma_{1}$ when the dissipation factor is small, such as when $\varepsilon_{1}$ is large. To verify this conclusion, the phase wavefront based on the Fermat principle and the COMSOL-simulated travel time are derived using the procedure in Table 2 and compared with the theory. Once the wave propagation paths are determined, the incident angles in medium#2 ($∠\beta_{in}$) and the refractive angles in medium#1 (∠$\beta_{out}$) for each of the receiving antennas are calculated, and the results are shown in Figure 10.

The results in Figure 10 indicate that the calculated direction of the phase wavefront derived from COMSOL (simulated travel time) and theory (phase distribution as per (9)) aligns well with those predicted by the Fermat principle when the matching medium (medium#1) is lossless. In addition, changes in the conductivity of the matching medium have a small influence on the phase wavefront when the permittivity of that medium is large. When $\varepsilon_{1}$ is relatively small, changes in the conductivity of the matching medium significantly change the incident and refractive angles. In this case, the angles calculated using the Fermat principle give a wrong estimation and far different values from the simulated travel time and theory (phase distribution). Consequently, for tracking algorithms based on the phase difference of received signals, Fermat’s principle can only be used when the investigated media have a very low dissipation factor. However, the Fermat principle gives a completely wrong estimation for media with a high dissipation factor, such as bio tissues. In these cases, the phase wavefront calculated using the proposed method should be applied.

**Figure 11:**
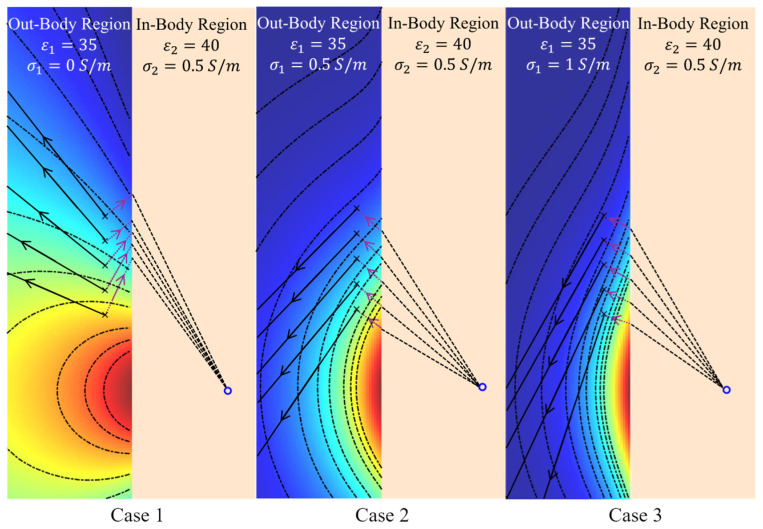
The amplitude wavefront of the electric field at 2.3 GHz in the out-of-body region when its conductivity ($\sigma_{1}$) varies from 0 S/m to 1 S/m (i.e., different matching media). The purple dot lines represent the directions of the amplitude attenuation, while the black solid lines represent the directions of the real-power-only flow.

It can be inferred from the previous results (Figure 4) that EM amplitude wavefront changes with variations in the medium’s loss in the matching medium. Applying (9) on the in-body communications model of Figure 8, the results of Figure 11 confirm the dependence of the amplitude wavefront on the medium’s loss. The effective direction of propagation, which is defined by the real-power-only direction (denoted as black solid lines in Figure 11), is shown to be orthogonal to the attenuation direction [39]. Consequently, variations in $\sigma_{1}$ may lead to different localization of the Tx antenna in algorithms based on signal amplitudes. To verify this hypothesis, the position shift of the Tx antenna shown in Figure 8 is calculated based on the compensated amplitude of the received signals. The amplitude compensation is performed using the signal received at the Rx antenna positioned opposite to the Tx antenna, where the line connecting the Tx and Rx is perpendicular to the in-body and out-of-body interface. Since the wave propagation path of this Tx/Rx pair is perpendicular to the interface, it remains constant despite variations in $\sigma_{1}$. Therefore, the signal loss caused by different values of $\sigma_{1}$ can be quantified. The procedure for signal amplitude compensation is summarized below:

- The signals from the selected Tx/Rx pair are simulated with $\sigma_{1}$ varying from 0 S/m to 0.7 S/m in increments of 0.1 S/m.
- The signal losses caused by different values of $\sigma_{1}$ (excluding the case when $\sigma_{1}=0$ S/m) are calculated based on the simulated signal amplitude at $\sigma_{1}=0$ S/m.
- The amplitudes of the received signals at all other Rx antennas are compensated using the signal losses calculated in Step 2.

**Figure 12:**
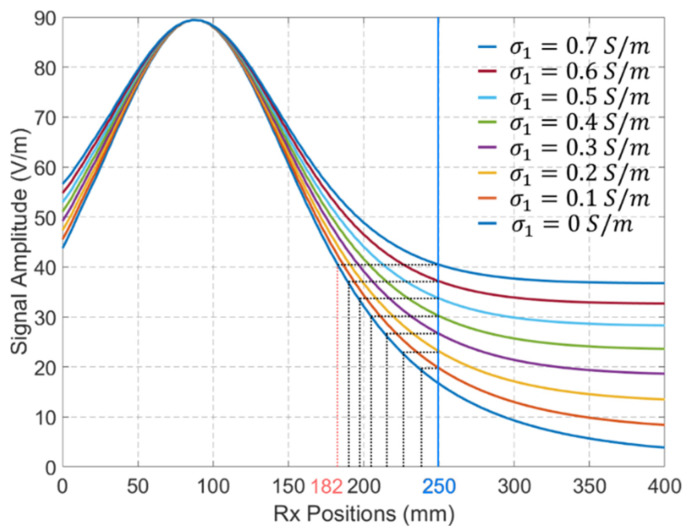
The compensated signals for EM propagation in media with different values of $\sigma_{1}$. Tx antenna’s position is defined by Figure 8.

Figure 12 shows the compensated signals for cases with different values of $\sigma_{1}$ when the Tx antenna is located at the position shown in Figure 8. The results indicate that changes in $\sigma_{1}$ lead to significant variations in the received signal distributions. For example, the received signal by the Rx located at 250 mm when $\sigma_{1}=0.7$ S/m is similar to the received signal by the Rx located at 182 mm when $\sigma_{1}=0$ S/m. Assume the position of Tx is derived based on the maximum amplitude of the received signal (with a higher signal amplitude indicating proximity to the Tx antenna), the maximum localization error for Rx antennas positioned between 250 mm and 400 mm is 68 mm when the received signal at $\sigma_{1}=0$ S/m is used as the reference signal.

**Figure 13:**
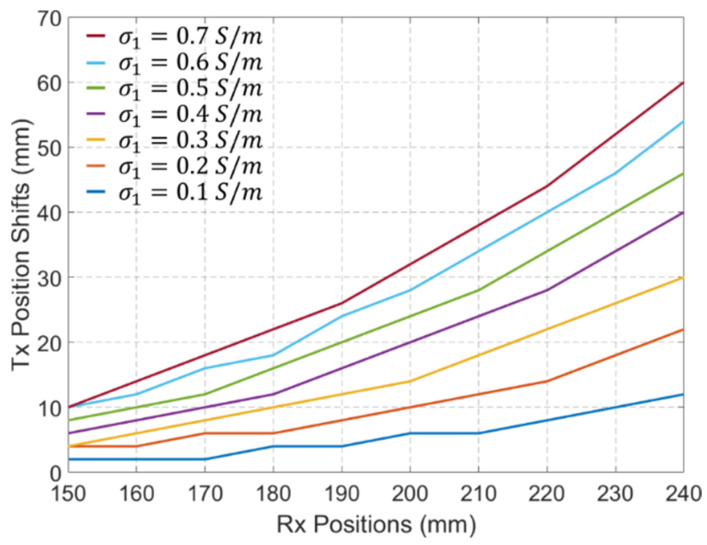
Tx position shifts in 10 Rx antennas evenly distributed between 150 mm and 240 mm when the values of $\sigma_{1}$ change from 0.1 S/m to 0.7 S/m.

To further quantify and visualize the localization error caused by different values of $\sigma_{1}$, the Tx position shifts in 10 Rx antennas evenly distributed between 150 mm and 240 mm are calculated and shown in Figure 13. An increase in the value of $\sigma_{1}$ leads to an increase in the localization error. Furthermore, higher localization errors are observed for Rx antennas located farther from the Tx antenna. This is because changes in the values of $\sigma_{1}$ result in a larger impact on the refractive angle of the signal amplitude at the Rx antennas located farther from the Tx, as presented in Figure 11.

## 4. Conclusions

This paper has investigated, using theory and numerical simulations, the impact of the dual EM wavefronts in lossy tissues on medical EM imaging, sensing, and in-body communications. It is known that a uniform EM wave traveling from an imaging antenna through a lossy matching medium inside the tissue has two distinct wavefronts: amplitude and phase. For media with a very low dissipation factor, it might be possible to estimate the phase wavefront and travel time inside tissues using the Fermat principle. However, for media with a higher dissipation factor, the Fermat principle fails to accurately predict the phase wavefront direction, leading to incorrect travel time estimations. These factors are crucial for developing processing algorithms in medical EM imaging that rely on wave travel time or phase changes to image tissues. Additionally, the results highlight the significant impact of the dielectric properties of the lossy matching medium on the amplitude wavefront, affecting the performance of amplitude-based processing algorithms, such as those used in wireless endoscopy.

## Figures and Tables

**Table 1:** The dielectric properties of samples of biological tissues across 0.5–4 GHz [49,50,51].

Tissue	Skin	Fat	Muscle	Blood	Bone	Tumor	Stomach	Brain
$\epsilon_{r}$@(0.5–4 GHz)	45–36.6	11.5–10.4	56.4–50.8	63.3–55.7	12.9–10.6	~75–70	66.7–60	53.7–42.4
σ (s/m)@(0.5–4 GHz)	0.72–2.34	0.08–0.05	0.82–3.02	1.38–4.13	0.1–0.7	0.05–3.5	1–3.85	1.08–3.28

**Table 2:** The procedures to calculate the wavefront using the Fermat principle and the signal travel time.

* 1. Define* M *evenly distributed points on the interface between the in-body and out-of-body regions. The interval between these points is 2 mm.* *2. Calculate the wave speed in the out-of-body region* $v_{1}$ *and in-body region* $v_{2}$. *3. Calculate the distance between the Tx and each entry point (*$d_{tx,m}$*) and the distance between each Rx and each entry point (*$d_{rx,m}$*)*. *4. Define an empty set *P=()* for the index of refractive points on the interface* Derive the wave path based on the Fermat principle: *5. for rx = 1 : N* * for m = 1 : M* $t(m)=d_{tx,m}/v_{2}+d_{rx,m}/v_{1}$ *end* $P\left(rx\right)=argmin(t)$ *end* *6. Connect the Tx and Rx points to each of the refractive points in * P * to form the wave paths.* Derive the wavefront based on the signal travel time from COMSOL: *7. Calculate the signal travel time from the Tx to each of the Rx, denoted as* ${∆t}_{tx,rx}$*, using the time delay of the maximum signal intensities of the Tx and Rx time-domain signals.* *8. for rx = 1 : N* *for m = 1 : M* $t(m)=d_{tx,m}/v_{2}+d_{rx,m}/v_{1}$ *end* $P\left(rx\right)=argmin(\left|{∆t}_{tx,rx}-t\right|)$ *end* *9. Connect the Tx and Rx points to each of the refractive points in* P *to form the wavefront.*

## Data Availability

All the data has been included in the article.

## References

[1] Mehrotra P., Chatterjee B., Sen S. (2019). EM-Wave Biosensors: A Review of RF, Microwave, mm-Wave and Optical Sensing. Sensors.

[2] Chen Z.Y., Gao Y.M., Du M. (2018). Propagation characteristics of electromagnetic wave on multiple tissue interfaces in wireless deep implant communication. IET Microw. Antennas Propag..

[3] Michaelson S.M., Lin J.C. (1987). Propagation and Absorption in Tissue Media.

[4] Mateen H., Basar R., Ahmed A.U., Ahmad M.Y. (2017). Localization of Wireless Capsule Endoscope: A Systematic Review. IEEE Sens. J..

[5] Ali M.A., Tom N., Alsunaydih F.N., Yuce M.R. (2025). Recent Advancements in Localization Technologies for Wireless Capsule Endoscopy: A Technical Review. Sensors.

[6] Blanco-Angulo C., Martínez-Lozano A., Gutiérrez-Mazón R., Juan C.G., García-Martínez H., Arias-Rodríguez J., Sabater-Navarro J.M., Ávila-Navarro E. (2022). Non-Invasive Microwave-Based Imaging System for Early Detection of Breast Tumours. Biosensors.

[7] Friedrich C., Bourguignon S., Idier J., Goussard Y. (2020). Three-Dimensional Microwave Imaging: Fast and Accurate Computations with Block Resolution Algorithms. Sensors.

[8] Ahdi Rezaeieh S.A., Darvazehban A., Janani A.S., Abbosh A.M. (2021). Electromagnetic Torso Scanning: A Review of Devices, Algorithms, and Systems. Biosensors.

[9] Guha S., Jamal F.I., Wenger C. (2017). A Review on Passive and Integrated Near-Field Microwave Biosensors. Biosensors.

[10] Kiourti A., Abbosh A.M., Athanasiou M., Björninen T., Eid A., Furse C., Ito K., Lazzi G., Manoufali M., Pastorino M. (2022). Next-Generation Healthcare: Enabling Technologies for Emerging Bioelectromagnetics Applications. IEEE Open J. Antennas Propag..

[11] Sultan K., Abbosh A. (2023). Advancing Wearable Electromagnetic Knee Imaging: A Comprehensive Review of Systems, Frameworks, Key Challenges, and Future Directions. IEEE J. Electromagn. RF Microw. Med. Biol..

[12] Guo L., Alqadami A.S.M., Abbosh A. (2023). Stroke Diagnosis Using Microwave Techniques: Review of Systems and Algorithms. IEEE J. Electromagn. RF Microw. Med. Biol..

[13] Origlia C., Rodriguez-Duarte D.O., Tobon Vasquez J.A., Bolomey J.-C., Vipiana F. (2024). Review of Microwave Near-Field Sensing and Imaging Devices in Medical Applications. Sensors.

[14] Rezaeieh S.A., Darvazehban A., Khosravi-Farsani M., Abbosh A.M. (2021). Body-Matched Gradient Index Lens Antenna for Electromagnetic Torso Scanner. IEEE Trans. Antennas Propag..

[15] Abbosh Y.M., Sultan K., Guo L., Abbosh A. (2025). Non-Uniform Antenna Array for Enhanced Medical Microwave Imaging. Sensors.

[16] Mondal S., Srivastava A., Mukhopadhyay S. (2025). Thermoelastic wave propagation and reflection in biological tissue under nonlocal elasticity and Moore–Gibson–Thompson heat conduction: Modeling and analysis. Z. Für Angew. Math. Und Phys..

[17] Essa A., Almajali E., Mahmoud S., Amaya R.E., Alja’Afreh S.S., Ikram M. (2024). Wireless Power Transfer for Implantable Medical Devices: Impact of Implantable Antennas on Energy Harvesting. IEEE Open J. Antennas Propag..

[18] Shon A., Chu J.-U., Jung J., Kim H., Youn I. (2017). An Implantable Wireless Neural Interface System for Simultaneous Recording and Stimulation of Peripheral Nerve with a Single Cuff Electrode. Sensors.

[19] Nelson B.D., Karipott S.S., Wang Y., Ong K.G. (2020). Wireless Technologies for Implantable Devices. Sensors.

[20] Soliman M.M., Chowdhury M.E.H., Khandakar A., Islam M.T., Qiblawey Y., Musharavati F., Zal Nezhad E. (2021). Review on Medical Implantable Antenna Technology and Imminent Research Challenges. Sensors.

[21] RamRakhyani A.K., Mirabbasi S., Chiao M. (2011). Design and Optimization of Resonance-Based Efficient Wireless Power Delivery Systems for Biomedical Implants. IEEE Trans. Biomed. Circuits Syst..

[22] Ali M.A., Alsunaydih F.N., Rathnayaka A., Yuce M.R. (2024). Implementing an Autonomous Navigation System for Active Wireless Capsule Endoscopy. IEEE Sens. J..

[23] Kim J., Lee H.S., Hoang M.C., Jeong S., Kim J.S., Lee C., Kang B., Lee J., Son Y.D., Bang S. (2022). Redundant Electromagnetic Control of an Endoscopic Magnetic Capsule Driven by Multiple Electromagnets Configuration. IEEE Trans. Ind. Electron..

[24] Chen W., Sui J., Wang C. (2022). Magnetically Actuated Capsule Robots: A Review. IEEE Access.

[25] Zhang P., Xu Y., Chen R., Dong W., Li Y., Yu R., Dong M., Liu Z., Zhuang Y., Kuang J. (2022). A Multimagnetometer Array and Inner IMU-Based Capsule Endoscope Positioning System. IEEE Internet Things J..

[26] Zhang Q., Li Y., Xu H., Li X., Zhang X. (2023). Magnetic Localization Method of Capsule Endoscope Based on Hybrid Model. IEEE Trans. Instrum. Meas..

[27] Shao G., Tang Y., Tang L., Dai Q., Guo Y.X. (2019). A Novel Passive Magnetic Localization Wearable System for Wireless Capsule Endoscopy. IEEE Sens. J..

[28] Rahimi Sardo F., Rayegani A., Matin Nazar A., Balaghiinaloo M., Saberian M., Mohsan S.A., Alsharif M.H., Cho H.-S. (2022). Recent Progress of Triboelectric Nanogenerators for Biomedical Sensors: From Design to Application. Biosensors.

[29] Carcione J.M., Ursin B. (2016). On Fermat’s principle and Snell’s law in lossy anisotropic media. Geophysics.

[30] Adler R.B., Chu L.J., Fano R.M. (1968). Electromagnetic Energy Transmission and Radiation.

[31] Rashed R. (1990). A Pioneer in Anaclastics: Ibn Sahl on Burning Mirrors and Lenses. Isis.

[32] Allwright D. (2022). From Snell’s law to Fermat’s principle. J. Sound Vib..

[33] Radcliff R.D., Balanis C.A. (1982). Modified Propagation Constants for Nonuniform Plane Wave Transmission through Conducting Media. IEEE Trans. Geosci. Remote Sens..

[34] Guimarães L.G., Sampaio E.E.S. (2008). A note on Snell laws for electromagnetic plane waves in lossy media. J. Quant. Spectrosc. Radiat. Transf..

[35] Vorst A.V., Rosen A., Kotsuka Y. (2006). RF/Microwave Interaction with Biological Tissues.

[36] Roy J.E. (2003). New results for the effective propagation constants of nonuniform plane waves at the planar interface of two lossy media. IEEE Trans. Antennas Propag..

[37] Holmes J., Balanis C. (1978). Refraction of a uniform plane wave incident on a plane boundary between two lossy media. IEEE Trans. Antennas Propag..

[38] Zhang S., Liu L., Liu Y. (2020). Generalized laws of Snell, Fresnel and energy balance for a charged planar interface between lossy media. J. Quant. Spectrosc. Radiat. Transf..

[39] Frezza F., Tedeschi N. (2012). On the electromagnetic power transmission between two lossy media: Discussion. J. Opt. Soc. Am. A.

[40] Ulaby F.T., Ravaioli U. (2022). Fundamentals of Applied Electromagnetics.

[41] Oloumi D., Winter R.S.C., Kordzadeh A., Boulanger P., Rambabu K. (2019). Microwave Imaging of Breast Tumor using Time-Domain UWB Circular-SAR Technique. IEEE Trans. Med. Imaging.

[42] Aldhaeebi M.A., Alzoubi K., Almoneef T.S., Bamatraf S.M., Attia H., Ramahi O.M. (2020). Review of Microwaves Techniques for Breast Cancer Detection. Sensors.

[43] Wang L. (2018). Microwave Sensors for Breast Cancer Detection. Sensors.

[44] Sultan K. (2022). Design and Implementation of Electromagnetic Knee Imaging Systems. Ph.D. Thesis.

[45] Sultan K., Abbosh A. (2024). On-Body Cavity-Backed Slot Antenna With Pattern and Polarization Diversity for Medical Imaging. IEEE Trans. Antennas Propag..

[46] Mousavi S.M.H., Moosazadeh M., Guo L., Abbosh A.M. (2024). Compact Dual-Polarized Cavity-Backed Antenna With Wideband Performance for Deep Torso Imaging. IEEE Trans. Antennas Propag..

[47] Rezaeieh S.A., Tan Y.Q., Abbosh A., Antoniades M.A. Equivalent circuit model for finding the optimum frequency range for the detection of heart failure using microwave systems. Proceedings of the 2013 IEEE Antennas and Propagation Society International Symposium (APSURSI).

[48] Abbosh A., Bialkowski K., Guo L., Al-Saffar A., Zamani A., Trakic A., Brankovic A., Bialkowski A., Zhu G., Cook D. (2024). Clinical electromagnetic brain scanner. Sci. Rep..

[49] Dielectric Properties of Human Tissue. https://itis.swiss/virtual-population/tissue-properties/database/tissue-frequency-chart/.

[50] Gabriel S. (1996). The dielectric properties of biological tissues: iii. parametric models for the dielectric spectrum of tissues. Phys. Med. Biol..

[51] Gabriel S. (1996). The dielectric properties of biological tissues: ii. measurements in the frequency range 10 hz to 20 ghz. Phys. Med. Biol..

[52] Federal Communications Commission (2012). Medical Area Body Network. Final rule. Fed Regist.

[53] Meaney P.M., Pendergrass S.A., Fanning M.W., Paulsen K.D. (2003). Importance of Using a Reduced Contrast Coupling Medium In 2D Microwave Breast Imaging. J. Electromagn. Waves Appl..

